# Surgical resection of hepatic and rectal metastases of pancreatic acinar cell carcinoma (PACC): a case report

**DOI:** 10.1186/s12957-018-1457-8

**Published:** 2018-08-03

**Authors:** Yusuke Ohara, Tatsuya Oda, Tsuyoshi Enomoto, Katsuji Hisakura, Yoshimasa Akashi, Koichi Ogawa, Yohei Owada, Yu Domoto, Yoshihiro Miyazaki, Osamu Shimomura, Masanao Kurata, Nobuhiro Ohkohchi

**Affiliations:** Department of Gastrointestinal and Hepato-Biliary-Pancreatic Surgery, Faculty of Medicine, University of Tsukuba, 1-1-1 Tennodai, Tsukuba, Ibaraki 305-8575 Japan

**Keywords:** Pancreatic cancer, Acinar cell carcinoma, Rectal metastasis

## Abstract

**Background:**

Pancreatic acinar cell carcinoma (PACC), a rare variant of pancreatic malignancy, is generally managed the same way as pancreatic ductal adenocarcinoma (PDAC). Surgical resection is the gateway to curing it; however, once it metastasizes (usually to the liver, lungs, lymph nodes, or peritoneal cavity), systemic chemotherapy has been the only option, but with unfavorable results.

**Case presentation:**

A 67-year-old man with symptoms of loss of appetite and weight underwent surgery for malignancy of the pancreatic tail extending into the entire pancreas. The pathological diagnosis was PACC following total pancreatectomy. Twenty-four months after the pancreatectomy, a solitary liver metastasis was treated by partial hepatectomy, and, subsequently, 4 months later, he presented with melena. Further examination revealed a type-2 rectal tumor. Histological examination following biopsy revealed it to be rectal metastasis of PACC, and it was treated by abdominoperineal resection. Subsequently, the patient did not have tumor recurrence as of 40 months after pancreatectomy.

**Conclusions:**

This is a rare case of PACC presenting with metachronal metastases in the liver and rectum, and we successfully treated them by surgical resections. Since the malignant behavior of PACC is usually less than that of PDAC, surgical resection could be an option even for metastatic lesions when the number and extent of metastases are limited.

## Background

Pancreatic acinar cell carcinoma (PACC) is relatively rare; it accounts for 1–2% of pancreatic malignancies [1, 2]. The histological presentation of PACC is quite unique. It is composed of relatively uniform tumor cells arranged in acinar, glandular, trabecular, and solid structures, without ductal formation that is seen in pancreatic ductal adenocarcinoma (PDAC) [3]. The clinical prognoses of patients with PACC are usually much better than those of patients with PDAC, although approximately half the patients with PACC are metastatic at the time of diagnosis [4].

Despite these differences between PACC and PDAC, the clinical management is usually same in both. Surgical resection is the only potentially curative treatment for primary PACC, and chemotherapy or radiotherapy has been performed for locally advanced or metastatic PACC [5]. However, their therapeutic efficacies in metastatic PACC have not been established due to small sample sizes [6].

Here, we report a case of PACC with unusual metachronal metastases in the liver and rectum after 24 and 28 months of the initial curative pancreatectomy, respectively. Metastases of PACC are commonly seen in the liver and lymph nodes, whereas colorectal metastasis is atypical and extremely rare. We chose surgical resection for both liver and rectal metastases with satisfactory outcomes.

## Case presentation

**Fig. 1:**
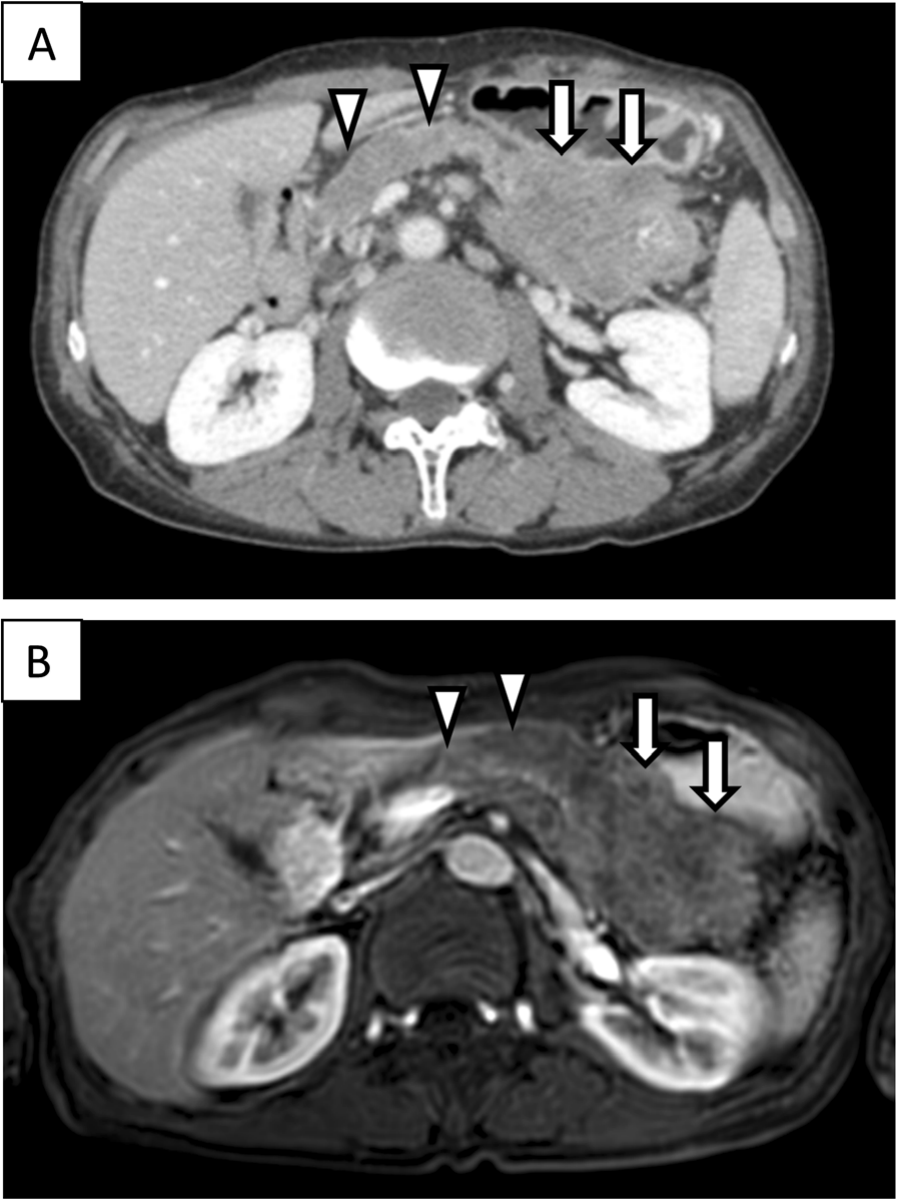
Episode 1: primary pancreatic tumor. Diffuse pancreatic mass in a 67-year-old man. Contrast-enhanced computed tomography (CT, **a**) scan and magnetic resonance imaging (MRI, **b**) showed a hypovascular pancreatic mass located mainly in the pancreatic tail (arrows) and involving the entire pancreas (arrow head)

**Fig. 2:**
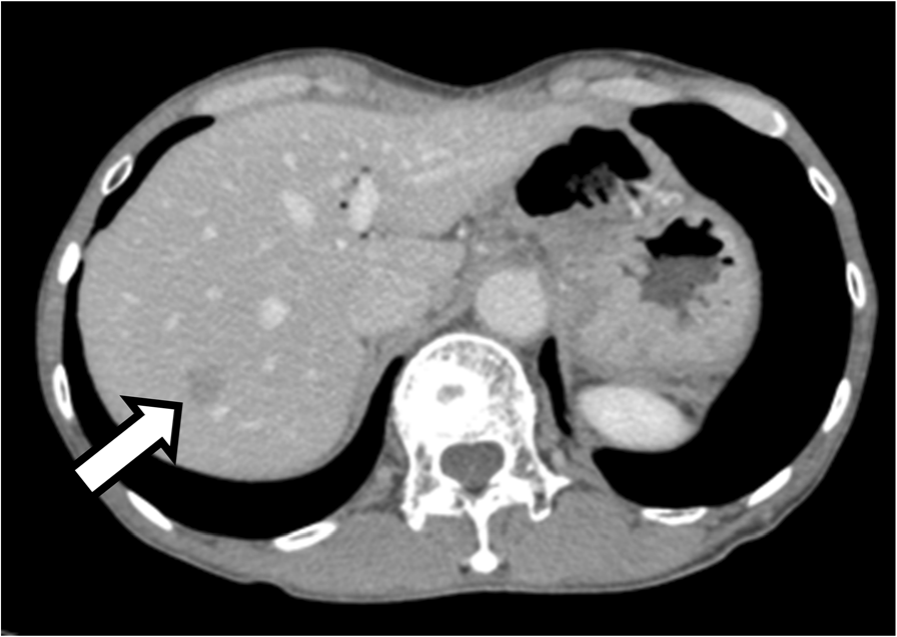
Episode 2: hepatic metastasis. Twenty-four months after the pancreatectomy. Contrast-enhanced computed tomography (CT) scan showed a solitary 1.5-cm hypovascular nodule in segment 7 of the liver (arrow)

**Fig. 3:**
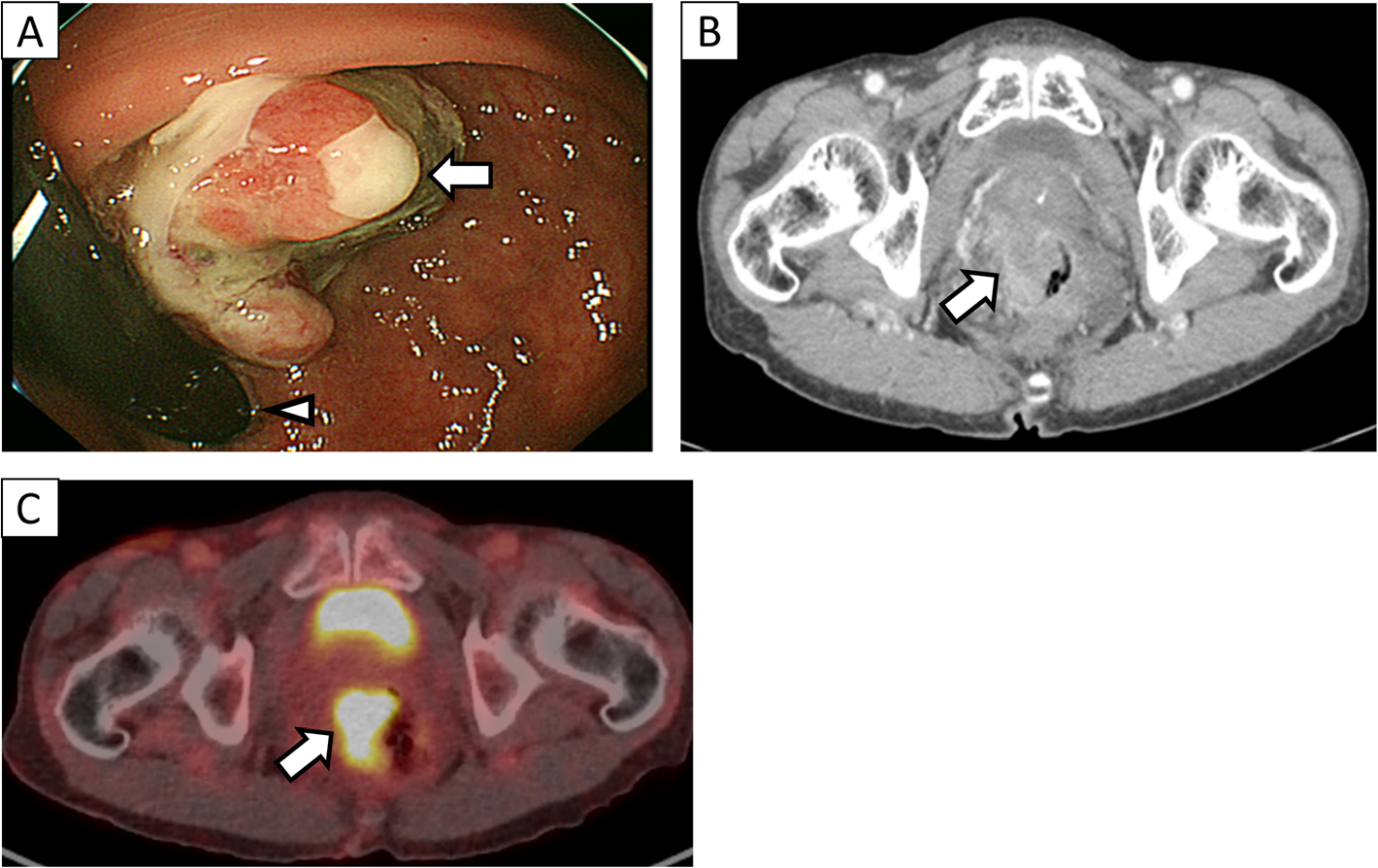
Episode 3: rectal metastasis. Twenty-eight months after the pancreatectomy. **a** Colonoscopy showed type-2 tumor in the lower rectum, which was located 1 cm above the dentate line. Histopathological examination following biopsy revealed it to be metastasis of PACC. Arrow, rectal tumor; arrow head, dentate line. **b** Computed tomography (CT) scan showed that the rectal tumor was 2.6 cm in diameter and slightly enhanced with the contrast agent (arrow). **c** Positron-emission tomography (PET) showed abnormal uptake of nuclear agent at the rectal tumor (arrow)

**Fig. 4:**
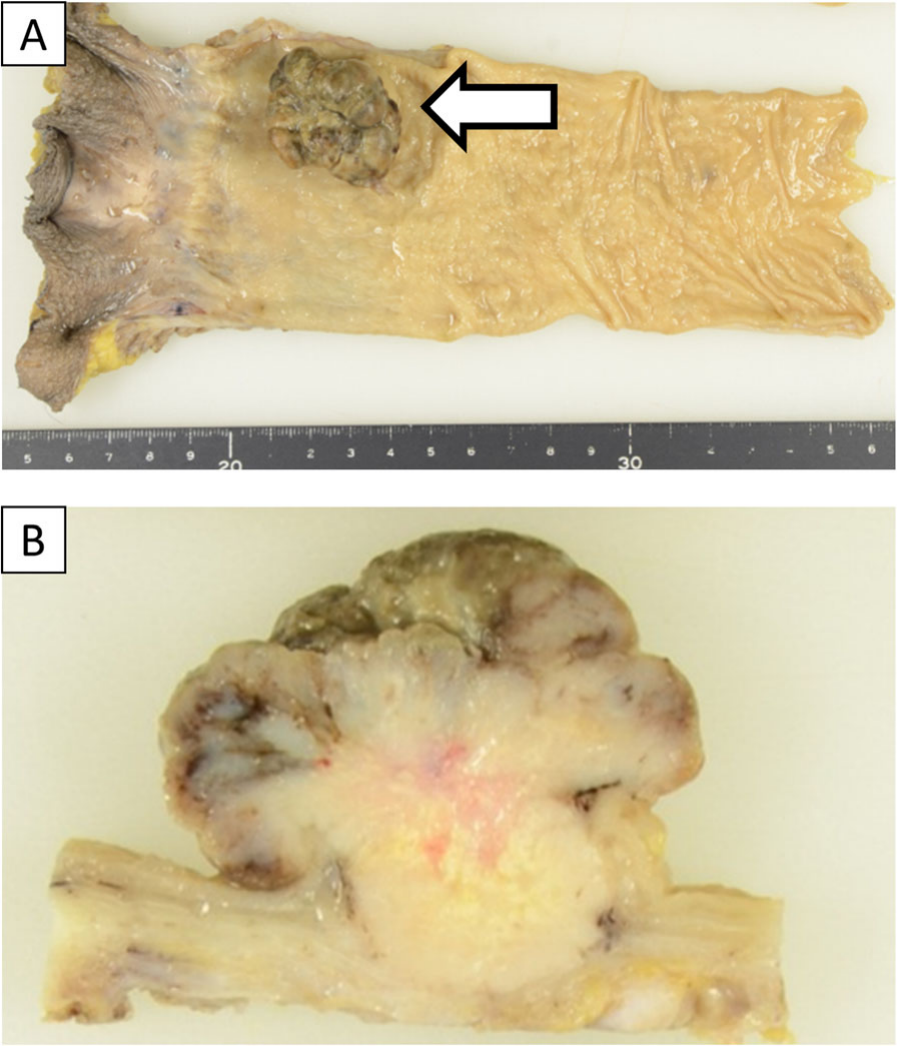
Gross features of the surgical specimen of the rectal tumor (**a**, frontal view). The cross-sectional loupe view demonstrated that the tumor created polypoid elevation of its surface, contained nodular components and ulceration, and penetrated the rectal mucosa into the submucosa and muscularis propria (**b**)

**Fig. 5:**
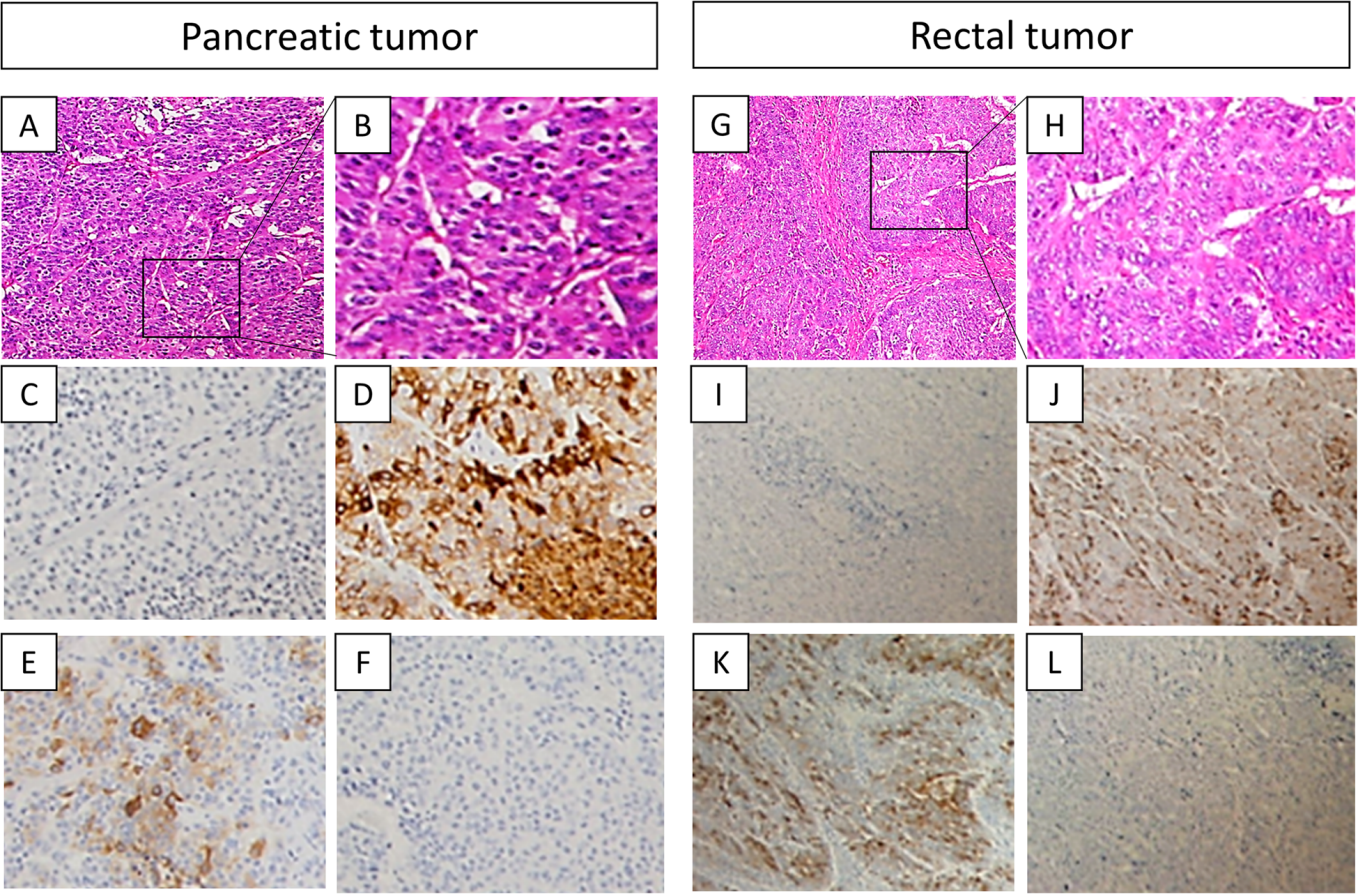
Histological examination of the pancreatic tumor (**a**–**f**) and rectal tumor (**g**–**l**). Relatively uniform tumor cells were found arranged in an acinar and solid formation. Both specimens showed the same morphological and immunohistochemical pattern. **a**, **g** Hematoxylin-eosin stain (H&E). **b**, **h** Magnified H&E image. **c**, **i** Negative for CDX2. **d**, **j** Positive for CK7. **e**, **k** Positive for CK19. **f**, **l** Negative for CK20

A 67-year-old man was admitted to the hospital with symptoms of loss of appetite and weight. Computed tomography (CT) and magnetic resonance imaging (MRI) revealed a pancreatic mass extending into the entire pancreas, splenic vein, and inferior mesenteric vein (Fig. 1). The patient underwent total pancreatectomy. Macroscopically, a whitish tumor measuring 10 cm was found in the pancreatic tail and body. Microscopically, eosinophilic tumor cells were found in a trabecular acinar pattern. Immunohistochemical analysis was negative for synaptophysin, chromogranin A, CD56, and trypsin. Finally, we diagnosed it as PACC, T3N0M0 (TNM classification according to the Union for International Cancer Control). According to the protocol for advanced PDAC, adjuvant chemotherapy with S-1 (Taiho Pharmaceutical, Tokyo, Japan) was administered for 11 months after pancreatectomy, and, subsequently, it was stopped due to the side effects (diarrhea, oral mucositis, fatigue, and hand-foot syndrome). Twenty-four months after the pancreatectomy, a solitary mass measuring 1.5 cm was found in segment 7 of the liver on CT (Fig. 2). The patient underwent posterior liver segmentectomy with a histopathological diagnosis of liver metastasis of PACC. Twenty-eight months after the pancreatectomy, the patient developed melena. Colonoscopy revealed a type-2 tumor at the lower rectum (1 cm above the dentate line, Fig. 3a), and biopsy revealed it to be rectal metastasis of PACC. CT and positron-emission tomography (PET) demonstrated the rectal tumor and an enlarged lymph node near the inferior mesenteric artery (Fig. 3b, c). The patient underwent laparoscopic abdominoperineal resection. Peritoneal dissemination was not found intraoperatively. Macroscopically, the tumor was 4 cm long, created polypoid elevation of its surface, contained nodular components and ulceration, and penetrated the rectal mucosa into the submucosa and muscularis propria (Fig. 4). Histopathology showed severe nuclear atypia of the tumor cells, and immunohistochemical analysis using CDX2, cytokeratin (CK)7, CK19, and CK20 confirmed the same profile as that of the specimen from pancreatectomy (Fig. 5). One lymph node out of 32 contained metastases. Thus, the pathological diagnosis was rectal and lymph node metastasis of PACC. All three surgical operations resulted in no severe postoperative complications. Periodic radiological examinations showed no tumor recurrence at 40 months after the pancreatectomy without additional chemotherapy. Our clinical decision was approved by the cancer board that included surgeons, oncologists, radiologists, and pathologists at the University Hospital of Tsukuba. Informed consent was obtained from the patient.

## Discussions and conclusions

PACC, a rare pancreatic cancer, demonstrates different behaviors and outcomes in comparison with PDAC. PDAC is the most common type of pancreatic cancer and is fatal in almost all cases. Frequently, it extends and/or metastasizes to the vessels, perineural tissue, adjacent organs, lymph nodes, liver, lungs, bones, and adrenals [7–10]. In contrast, though PACC is also invasive, it is less aggressive than PDAC [4, 11]. Schmidt et al. compared 865 cases of PACC with 367,999 of PDAC and demonstrated that the stage-specific 5-year survival rate in PACC is better than that in PDAC (e.g., PACC 40.2% vs. PDAC 9.8% at stage II) [12]. Wisnoski et al. showed that the median survival time in PACC was 47 months, whereas it was only 4 months in PDAC [13]. As in PDAC, surgical resection should be performed in PACC with a goal of achieving R0 margins [12]. For metastatic PACC, the chemotherapy protocol of PDAC has been used, which includes gemcitabine, 5-FU, oxaliplatin, CPT-11, and S-1, or their combinations [14–16]. A systematic review by Glazer et al. demonstrated that the disease control rate of chemotherapy was 55% and median survival time in metastatic PACC after chemotherapy was 17 months [17]. However, the efficacies of various chemotherapies have not been studied in controlled, prospective studies, and there are no definitive guidelines for the treatment of metastatic PACC. Additionally, as Abraham et al. demonstrated, PACC shows different clinicopathological and genetic features from PDAC [18]. Therefore, strategies other than chemotherapy may be considered for metastatic PACC.

Resection of metastases in PACC, as performed in this case, is not routinely performed [17]. We chose resection of the liver metastases for two reasons. First, liver metastases are often multiple in PACC [19]; however, in this case, it was solitary, slow growing, and relatively small even 24 months after the primary pancreatectomy. Second, additional chemotherapy was not favorable for this patient due to the severe side effects of the previous treatment with S-1. After resection of hepatic metastasis, the rectal metastasis including mesorectal lymph node metastasis was found, and we estimated that it could be removed completely with additional resection, which would also be effective in preventing rectal bleeding or obstruction. Would these surgical resections be widely applicable to other cases of PACC? Hartwig et al. surveyed six cases of metastatic PACC and presented the effectiveness of surgical resection in these cases with limited metastases [20]. They studied the long-term survival of patients with synchronous or metachronous metastatic disease and non-metastatic disease who underwent resection and found no significant differences between the two groups with 2-year survival rates of 67% and 69%, respectively. We believe that potentially resectable metastases in PACC can be treated by surgical resection, as long as the surgery is associated with low morbidity.

The colon and rectum are quite rare sites of metastases from pancreatic cancer, as only a few such cases have been reported [21–23]. As an example, Ogu et al. reported a case of metachronous sigmoid colon metastasis of PDAC 24 months after pancreatectomy, which was treated by colectomy [23]. However, to the best of our knowledge, there are no reports of colorectal metastasis in PACC. As suggested by earlier studies, we performed immunohistochemical analysis including testing for CK7 and CK20 to confirm that the rectal tumor showed the same profiles as those of the primary PACC and that it was different from other rectal malignancies [23, 24]. It should be noted that acinar cell carcinoma would primarily occur in the colon and rectum as reported in several studies, in which the patients did not have any pancreatic tumors [25, 26]. In our case, a large invasive pancreatic tumor had presented 28 months before the detection of the rectal tumor; therefore, we consider the rectal tumor as not a primary tumor but rather a metastasis of PACC. The possibility that this rectal tumor was secondary to peritoneal dissemination was rejected since the peritoneum including the Douglas pouch was clear, and the tumor was located mainly in the muscular and mucosal parts of the rectal wall while the adventitia was free from the tumor. We suspected that the colorectal metastasis might have developed via the hematogenous pathway due to severe vascular invasion of the primary pancreatic cancer followed by metastasis of the mesorectal lymph nodes; however, the true mechanism of rectal metastasis could not be demonstrated.

We presented a rare case of metachronous hepatic and rectal metastases of PACC treated with surgical resection. Our case indicates that aggressive and curative surgery can be an option in metastatic PACC. The therapeutic strategy against metastases from “less invasive” PACC should include surgical resection in addition to chemotherapy to improve the prognosis.
